# Respiratory Syncytial Virus Infection of Human Lung Fibroblasts Induces a Hyaluronan-Enriched Extracellular Matrix That Binds Mast Cells and Enhances Expression of Mast Cell Proteases

**DOI:** 10.3389/fimmu.2019.03159

**Published:** 2020-01-28

**Authors:** Stephen R. Reeves, Kaitlyn A. Barrow, Lucille M. Rich, Maria P. White, Nicholas J. Shubin, Christina K. Chan, Inkyung Kang, Steven F. Ziegler, Adrian M. Piliponsky, Thomas N. Wight, Jason S. Debley

**Affiliations:** ^1^Division of Pulmonary and Sleep Medicine, Seattle Children's Hospital, Seattle, WA, United States; ^2^Center for Immunity and Immunotherapies, Seattle Children's Research Institute, Seattle, WA, United States; ^3^Department of Pediatrics, University of Washington, Seattle, WA, United States; ^4^Matrix Biology Program, Benaroya Research Institute, Seattle, WA, United States; ^5^Immunology Program, Benaroya Research Institute, Seattle, WA, United States

**Keywords:** airway inflammation, airway remodeling, extracellular matrix, human lung fibroblasts, hyaluronan, mast cells, respiratory syncytial virus, tumor necrosis factor-stimulated gene 6

## Abstract

Human lung fibroblasts (HLFs) treated with the viral mimetic polyinosine-polycytidylic acid (poly I:C) form an extracellular matrix (ECM) enriched in hyaluronan (HA) that avidly binds monocytes and lymphocytes. Mast cells are important innate immune cells in both asthma and acute respiratory infections including respiratory syncytial virus (RSV); however, the effect of RSV on HA dependent mast cell adhesion and/or function is unknown. To determine if RSV infection of HLFs leads to the formation of a HA-enriched ECM that binds and enhances mast cell activity primary HLFs were infected with RSV for 48 h prior to leukocyte binding studies using a fluorescently labeled human mast cell line (LUVA). Parallel HLFs were harvested for characterization of HA production by ELISA and size exclusion chromatography. In separate experiments, HLFs were infected as above for 48 h prior to adding LUVA cells to HLF wells. Co-cultures were incubated for 48 h at which point media and cell pellets were collected for analysis. The role of the hyaladherin tumor necrosis factor-stimulated gene 6 (TSG-6) was also assessed using siRNA knockdown. RSV infection of primary HLFs for 48 h enhanced HA-dependent LUVA binding assessed by quantitative fluorescent microscopy. This coincided with increased HLF HA synthase (HAS) 2 and HAS3 expression and decreased hyaluronidase (HYAL) 2 expression leading to increased HA accumulation in the HLF cell layer and the presence of larger HA fragments. Separately, LUVAs co-cultured with RSV-infected HLFs for 48 h displayed enhanced production of the mast cell proteases, chymase, and tryptase. Pre-treatment with the HA inhibitor 4-methylumbelliferone (4-MU) and neutralizing antibodies to CD44 (HA receptor) decreased mast cell protease expression in co-cultured LUVAs implicating a direct role for HA. TSG-6 expression was increased over the 48-h infection. Inhibition of HLF TSG-6 expression by siRNA knockdown led to decreased LUVA binding suggesting an important role for this hyaladherin for LUVA adhesion in the setting of RSV infection. In summary, RSV infection of HLFs contributes to inflammation via HA-dependent mechanisms that enhance mast cell binding as well as mast cell protease expression via direct interactions with the ECM.

## Introduction

Lower respiratory tract infections caused by respiratory syncytial virus (RSV) are the leading cause of hospital admissions for infants worldwide accounting for an estimated 3.4 million hospitalizations and leading to an estimated 239,000 deaths in children under the age of 5 years annually (1, 2). While these cases are generally related to the most severe episodes of RSV bronchiolitis, exposure to RSV is widespread with 50–65% of infants under the age of 1 year and nearly 100% of all children demonstrating evidence of prior RSV infection by the age of 2 years (3). In addition to the major public health concerns brought on by acute RSV infection in infancy, RSV infections have also been identified as a significant independent risk factor for the subsequent development of asthma, the most common chronic disease of childhood (4, 5).

Previous pathology specimen studies in humans have highlighted that ciliated bronchial epithelial cells (BECs) are the primary target for RSV lower respiratory tract infection; however, non-ciliated and non-epithelial cells also stain positively for RSV infection (6). Separate studies have identified a role for mesenchymal cells such as human lung fibroblasts (HLFs) in contributing to airway inflammatory changes in the setting of respiratory viruses, either by acting as an additional reservoir for replication or as an amplification site of proinflammatory signaling (7); however, the contributions of HLFs to the inflammatory response to RSV have yet to be elucidated *in vivo*. HLFs have been identified as an important regulator of the cellular microenvironment through the establishment of an extracellular matrix (ECM) that is permissive for inflammatory cell migration and enhances the retention of monocytes and lymphocytes at sites of inflammation (8–14). While several ECM constituents are likely to contribute to the establishment of a pro- or anti-inflammatory microenvironment, numerous studies have implicated the glycosaminoglycan hyaluronan (HA) as a significant contributor to the immunomodulatory functions of the ECM in both acute and chronic respiratory diseases (13, 15, 16).

Previous work has demonstrated that stimulation of HLFs with the viral mimetic, polyinosine-polycytidylic acid (poly I:C), a toll-like receptor 3 (TLR-3) agonist, led to the accumulation of an ECM that was enriched with HA and demonstrated an increased capacity to bind and retain monocytes in a HA-dependent manner (17). While these studies did not directly investigate the effects of HLF infection with RSV, the results of increased HA-dependent binding of monocytes and the establishment of a HA-enriched ECM within the cell layer were consistent with earlier studies that demonstrated intestinal smooth muscles stimulated with RSV or poly I:C produced a HA-enriched ECM that displayed enhanced binding of monocytes (18). Neither of these studies evaluated the subsequent effects on monocyte phenotype or cell signaling, thus the downstream effects of interacting with the HA-enriched ECM are unknown.

Mast cells are best characterized for their role in atopy and asthma; however, their contribution to the innate immune response to viral infection is of increasing interest (19). For example, recent studies have demonstrated that mast cell-derived serine protases are able to modulate innate immune signaling from epithelial cells leading to enhanced inflammatory responses (20). Mast cells are not typically present in the lungs of healthy children, but are recruited in the setting of respiratory viral infections, which may represent a link between early life respiratory infections and subsequent development of asthma (21). Furthermore, emerging evidence has demonstrated that mast cells express CD44, the major HA receptor, and that CD44 activity is critical for mast cell proliferation and differentiation (22, 23). To date, no studies investigating the interactions between HA and mast cells in the setting of RSV infection have been reported.

Based on the findings of these prior studies, we hypothesized that infection of primary healthy human HLFs with RSV would lead to the establishment of an HA-enriched ECM that would promote the retention of mast cells. Additionally, we hypothesized that HA-enriched ECM would promote protease expression by mast cells that were bound to the resultant ECM. To test these hypotheses, we employed a human mast cell line (LUVA) and examined the expression of mast cell proteases, which have been associated with proinflammatory mediators, following 48 h of co-culture on the RSV-induced ECM. Our findings demonstrate that RSV infection of healthy pediatric HLFs promotes the establishment of an ECM that is enriched with HA in the cell layer with enhanced capacity to adhere mast cells and enhance the production of mast cell proteases.

## Methods

### Cell Culture Models

Primary healthy pediatric HLFs were obtained from Lonza. Cells were expanded until passage #5 and used for all studies described herein. HLFs were seeded at a density of ~2,500 cells/cm^2^ in 12-well plates (Corning^®^ Life Sciences) and were maintained in 10% FBS DMEM supplemented with Pen/Strep and L-glutamine with media changes every 48 h. LUVA cells were generously provided by Dr. John Steinke, University of Virginia (24) and were maintained in culture flasks using Stempro-34 media (Thermo Fisher Scientific) supplemented with Pen/Strep and L-glutamine with media changes every 48–72 h. RSV (line 19, A strain) was obtained and propagated as described (25). Knockdown of tumor necrosis factor-stimulated gene 6 (TSG-6) in HLFs was achieved using TSG-6 siRNA (4392420, Silencer^®^ Select, Thermo Fisher) and Lipofectamine™ RNAiMAX Reagent (Thermo Fisher) according to the manufacturer's recommendations. Optimal transfection conditions were assessed based on manufacturer's suggested protocols leading to ~95% reduction of TSG-6 expression detected by RT-PCR. For TSG-6 siRNA knockdown experiments, control conditions included transfection of non-coding scrambled siRNA.

#### LUVA-HLF Co-cultures

Confluent HLF monolayers were established and were infected with RSV 48 h as described above prior to the initiation of LUVA-HLF cocultures. LUVA cells were added to the HLF cultures in 12-well plates (5 × 10^5^ cells per well) with a fresh media change. LUVA-HLF co-cultures with and without RSV infection were incubated for an additional 48 h after which LUVA cells were harvested for analysis as described below.

### Time Course for Experiments

To establish the optimal timeline for experiments, preliminary studies were performed to investigate the time course for mast cell binding at 24, 48, and 96 h (data not shown). Maximal mast cell binding following RSV infection of HLFs was demonstrated at the 48-h timepoint and was unchanged by the 96-h timepoint, thus all additional experiments were conducted at 48 h post-RSV infection.

The timeline for experiments included two different paradigms depending on outcome measures. For all experiments, HLFs were seeded and grown for 3 days to achieve confluence following which HLFs were infected with RSV (MOI = 1) at experimental Day 0. The first set of studies included endpoint LUVA cell adhesion assays after 48 h of HLF RSV infection. In addition to LUVA cell binding assays, samples were isolated at this point for gene expression analysis of HLFs and characterization of HA accumulation and fragment size. The second paradigm included LUVA cells co-cultured with RSV-infected HLFs at the 48-h timepoint and maintained in co-culture for an additional 48 h at which point LUVA cells were collected following a brief hyaluronidase (from *Streptomyces hyalurolyticus;* Catalog # H1136, MilliporeSigma) treatment to remove adherent LUVA cells from the HA-enriched ECM, leading to ~90% recovery of LUVA cells embedded in the HA-enriched ECM. HLFs and LUVA cell samples were collected and lysed for western blot. A subset of HLFs was treated with 2.5 mM 4-methylumbelliferone (4-MU; Catalog # M1381, MilliporeSigma), a HA synthase (HAS) inhibitor, at the time of RSV infection to inhibit formation of the HA-enriched ECM (26) and was re-dosed with each media change. In parallel, additional LUVA-HLF co-cultures were treated with monoclonal neutralizing antibodies against CD44 (30 μg/mL; Catalog # MA4400, Thermo Fisher) at the time of co-culture to block interactions between LUVAs and HA (27). A separate subset of HLFs was treated with siRNA to knockdown expression of TSG-6 24 h prior to RSV infection. LUVA cells were isolated following 48 h of co-culture for gene expression analysis, binding assays, and immunohistochemistry.

### RNA Extraction and Real-Time PCR

For gene expression analysis experiments, total RNA was isolated from either HLFs or LUVA cells according to manufacturer recommendations (RNAqueous kit, Ambion^®^-Applied Biosystems). RNA concentration and quality were determined using the NanoDrop™ One Microvolume UV-Vis Spectrophotometer (Thermo Fisher Scientific). RNA samples were reverse-transcribed using the SuperScript^®^ VILO cDNA Synthesis Kit (Life Technologies). Real-time PCR was performed using validated TaqMan^®^ probes (Life Technologies) for hyaluaronan synthase (HAS) 1, HAS2, HAS3, hyaluronidase (HYAL) 1, HYAL2, CD44, receptor for HA mediated motility (RHAMM), lymphatic vessel endothelial HA receptor 1 (LYVE-1), versican (VCAN), TSG-6, chymase, tryptase, and glyceraldehyde 3-phosphate dehydrogenase (GAPDH, see Table 1 for additional details). Assays were performed using the TaqMan^®^ Fast Advanced Master Mix reagents and the Applied Biosystems StepOnePlus™ Real-Time PCR System (Life Technologies).

**Table 1:** List of PCR primers.

**PCR Target**	**Gene ID**	**Assay ID**
Chymase	CMA1	Hs01095979_g1
Cluster of differentiation 44	CD44	Hs01075861_m1
Glyceraldehyde 3-phosphate dehydrogenase	GAPDH	Hs02758991_g1
Hyaluaronan synthase 1	HAS1	Hs00987418_m1
Hyaluaronan synthase 2	HAS2	Hs00193435_m1
Hyaluaronan synthase 3	HAS3	Hs00193436_m1
Hyaluronidase 1	HYAL1	Hs00201046_m1
Hyaluronidase 2	HYAL2	Hs01117343_g1
Lymphatic vessel endothelial hyaluronan receptor 1	LYVE1	Hs00272659_m1
Receptor for hyaluronan mediated motility	RHAMM	Hs00234864_m1
Tryptase	TPSB2	Hs02576518_gH
Tumor necrosis factor-inducible gene 6	TNFAIP6	Hs01113602_m1
Versican	VCAN	Hs00171642_m1

### Western Blots

Samples were lysed with 1× lysis buffer (MPER + 1X Halt protease inhibitor cocktail + 5 μM EDTA; Thermo Scientific, San Jose, CA, USA), and total protein concentration determined using the Pierce BCA protein assay kit (Thermo Scientific), according to the manufacturer's protocol. Equal amounts of protein were electrophoresed on 4–20% polyacrylamide gels and transferred onto PVDF membranes. Tryptase and glyceraldehyde 3-phosphate dehydrogenase (GAPDH) were detected with human anti-mast cell tryptase (clone G3, Santa Cruz Biotechnology, Santa Cruz, CA, USA) and human anti-GAPDH (clone GA1R, Cell Sciences) antibodies, respectively.

### HA Quantification and Characterization of Fragment Size

HA content and hydrodynamic size were assessed using a modification of reported methods (12, 28). Media and cell layer samples were isolated separately and digested with pronase (300 μg/ml, Roche) in 0.5 M Tris buffer (pH 6.5) for 18 h at 37°C. Following digestion, the pronase was heat inactivated by incubation at 100°C for 20 min. Media and cell layer concentrations of HA were measured using an Enzyme-Linked Immunosorbent Assay (ELISA) from R&D Systems^®^ (kit DY3614-05). To determine the hydrodynamic size of HA fragments contained within the samples, equal amounts of HA were applied to an S-1000 column (GE Healthcare) in a 0.5 M sodium acetate buffer containing 0.025% Chaps, 0.02% sodium azide at pH 7.0. Samples were collected using a microtube fraction collector (Model 2110, BioRad) and were analyzed separately using the HA ELISA described above.

### Mast Cell Binding Assays and Immunohistochemistry

Mast cell binding was assessed using a modification of reported methods (12, 18). Subsets of RSV-infected and control HLFs were used to assess the ability of the secreted ECM to bind LUVA cells. Parallel conditions pre-treated for 30 min at 37°C with hyaluronidase (4 U/mL; from *S. hyalurolyticus;* Catalog # H1136, MilliporeSigma) were included. LUVA cells were washed twice in phenol-free media and re-suspended (1 × 10^6^ cells/mL) and were then incubated with calcein-AM (0.5 μg/ml; Life Technologies) for 45 min at 37°C. HLF wells were washed with RPMI. Afterward, 1.0 mL of the mast cell suspension was added to the wells and allowed to bind at 4°C for 90 min to inhibit enzymatic HA turnover. Cultures were washed 5 times in cold RPMI to remove non-adherent cells. Adherent cell area was quantified using live-cell fluorescent microscopy (ImageXpress Pico, Molecular Devices). Following live-cell imaging, subsets of cells were fixed using a 10% formalin/70% ethanol/5% acetic acid fixative for 10 min at room temperature, washed with PBS, and stained with biotinylated hyaluronan binding protein (HABP) primary (2.5 μg/ml; Catalog # H0161, MilliporeSigma) and a streptavidin conjugated Alexa Fluor 568 secondary (1:1,000, Thermo Fisher). Plates were then reimaged to using the ImageXpress Pico to highlight interactions between the HA matrix and LUVA cells.

Separate HLF specimens were grown on sterilized, collagen coated 12 mm glass coverslips under the experimental conditions described above. Staining for HA was achieved as described above, HLF specimens were also stained with a primary antibody to inter-alpha-trypsin inhibitor heavy chain 1 (ITIH1, 1:500, Thermo Fisher) and a rabbit secondary antibody conjugated to Alexa Fluor 488 (1:1,000, Thermo Fisher). Imaging was performed by either conventional epi-fluorescence microscopy (DM6000B, Leica, Wetzlar, Germany) or confocal microscopy (TCS SP5, Leica).

### Statistical Analysis

Analyses of RT-PCR results were performed using GenEx version 6.0.5 (MultiD Analyses AB,) based on previously described methods (29). For all other data, the unpaired *t*-test was used for comparisons that were normally distributed within each group. For non-normally distributed data, the Mann-Whitney test was used. Statistical analyses were performed using Prism^®^ 8.0 software (Graph-Pad Software Inc.). Statistical significance was set at *P* < 0.05.

## Results

### RSV Infection Promotes Greater HA Synthesis by HLFs

**Figure 1:**
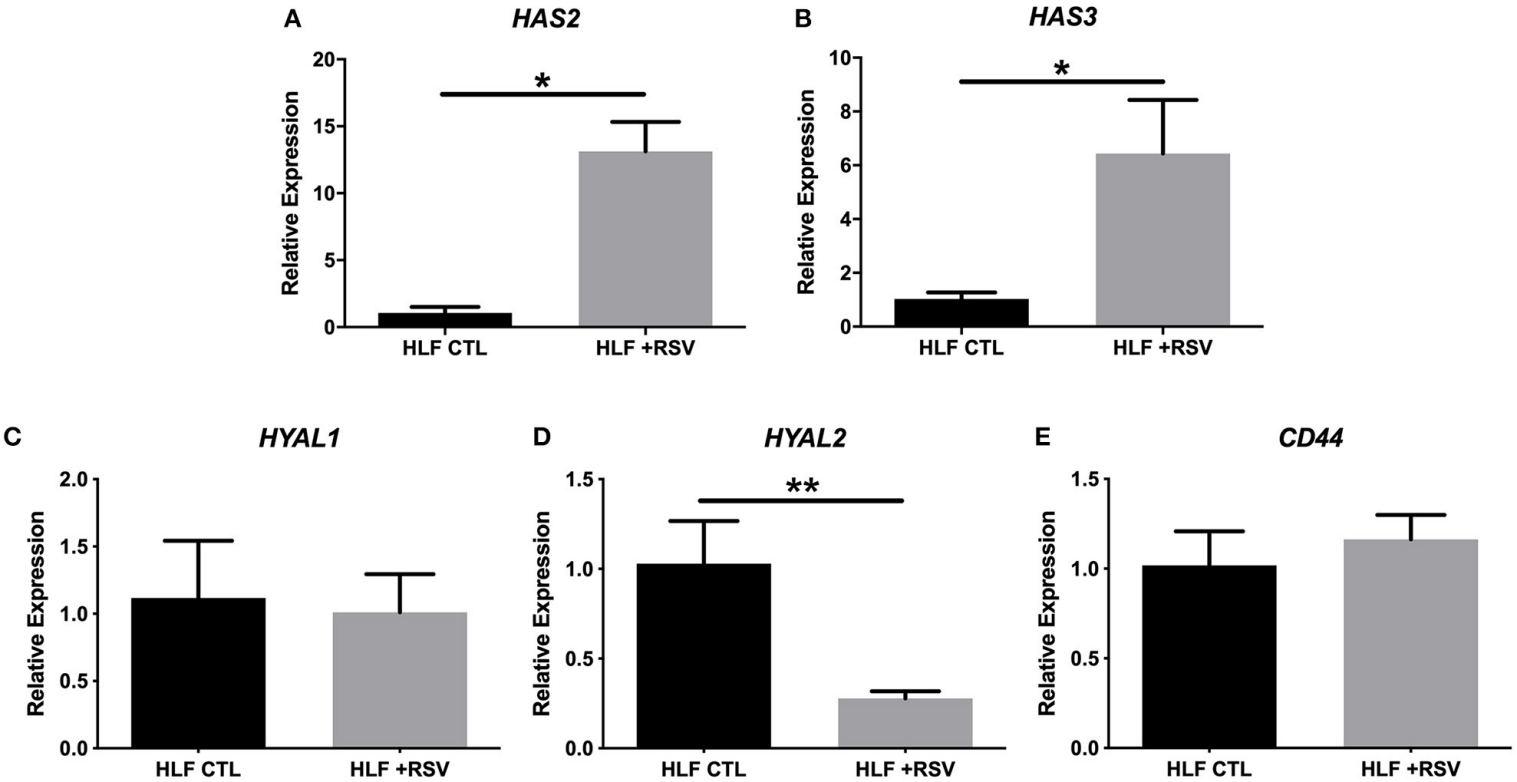
Expression of hyaluronan synthase (HAS) and hyaluronidase (HYAL) isoforms by human lung fibroblasts (HLFs) infected with respiratory syncytial virus (RSV). Expression of **(A)** *HAS2* and **(B)** *HAS3* were both significantly increased following RSV infection (13-fold, ^*^P < 0.0006 and 5.6-fold, ^*^P < 0.0006, respectively). HLF expression of **(C)** *HYAL1* was not significantly altered by RSV infection. Expression of **(D)** *HYAL2* was significantly decreased (~80% reduction, ^**^P < 0.0001) following RSV infection. Expression of CD44 was not significantly altered **(E)**. Gene expression was normalized to GAPDH and is shown as normalized mean ± SD relative to control HLFs for *N* = 7 replicates/group.

Comparison of gene expression by HLFs with or without RSV infection demonstrated enhanced expression of mRNA for HA synthetic enzymes (i.e., HAS isoforms) and decreased expression of mRNA for the HA degradation enzyme (*HYAL2*). HLF expression of *HAS1* was essentially undetectable in both control and RSV-infected HLFs, thus precluding statistical comparisons between the groups (not shown). Expression of *HAS2*, the major HAS isoform expressed by HLFs, was significantly elevated following the 48-h RSV infection (13-fold, *P* < 0.0006, Figure 1A) as was the expression of *HAS3* (5.6-fold, *P* < 0.0006, Figure 1B). To investigate the contributions of HA degradation mechanisms to the accumulation of HA, expression of *HYAL1, HYAL2*, and *CD44* mRNA transcripts was also assessed with and without RSV infection. No significant differences in *HYAL1* and *CD44* expression were found between RSV-treated or control HLFs (Figures 1C,E); however, expression of *HYAL2* mRNA transcripts by RSV-infected HLFs was significantly decreased (~80% decrease, *P* < 0.0001, Figure 1D).

**Figure 2:**
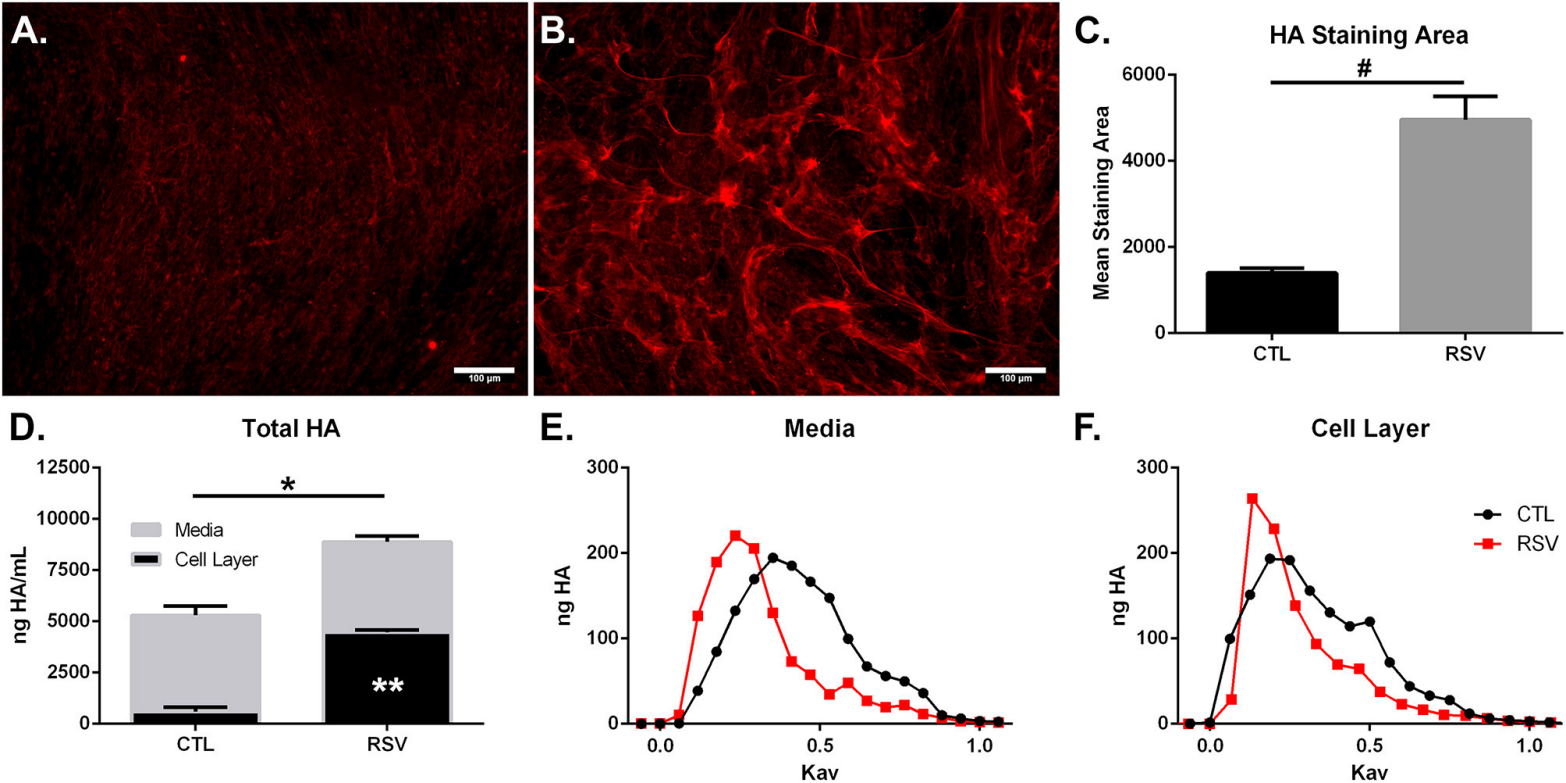
Imaging studies were performed to label HA deposition (red) by HLFs in **(A)** control HLFs and **(B)** RSV-infected HLFs. **(C)** Quantitative analysis of staining area demonstrated a 3.5-fold increase in the staining of HA (^#^*P* < 0.03). **(D)** Quantitative analysis of HA accumulation revealed that HLFs infected with RSV displayed increased total HA accumulation (8,878 ± 114.4 ng/mL vs. 5,295 ± 389.6 ng/mL, ^*^P < 0.0009). Differences were driven by increased HA contained in the HLF cell layer following RSV infection (4,477 ± 56.13 ng/mL vs. 600.8 ± 128.3 ng/mL, ^**^P < 0.0001) without significant differences in the media compartment HA content. Data shown as mean ± SD for *N* = 3 replicates/group. HLFs infected with RSV demonstrated a greater abundance of HMW-HA species compared to HLF controls in both **(E)** media and **(F)** cell layer compartments. A representative plot of HA profiles measured by S-1000 size exclusion chromatography is shown. Data is plotted as ng HA vs. the partition coefficient (K_av_) for *N* = 3 replicates/group.

In addition to characterization of gene expression of HA synthesis and degradation enzymes by HLFs, accumulation of HA in HLF cultures was assessed by immunofluorescence (Figures 2A,B). Staining for HA revealed an increase in HA accumulation in HLFs infected with RSV compared to control HLFs (Figure 2C; 3.5-fold increase, *P* < 0.03). Quantitative analysis of HA deposits assessed by ELISA demonstrated an increased total amount of HA contained within samples collected from RSV-infected HLFs compared to control HLFs (8,878 ± 114 ng/mL vs. 5,295 ± 389 ng/mL, *P* < 0.0009). This difference was driven by increased HA contained in the HLF cell layer following RSV infection (4,477 ± 56 ng/mL vs. 601 ± 128 ng/mL, *P* < 0.0001) without significant differences in the media compartment HA content (Figure 2D). In both the media and cell layer compartments, HA size was found to be skewed toward HMW-HA in RSV-infected samples compared to control HLFs with a more polydisperse pattern seen in the control samples (Figures 2E,F).

### HA-Enriched ECM Produced by RSV-Infected HLFs Promotes the Increased Adhesion of LUVA Cells as Well as the Expression of Mast Cell Proteases

**Figure 3:**
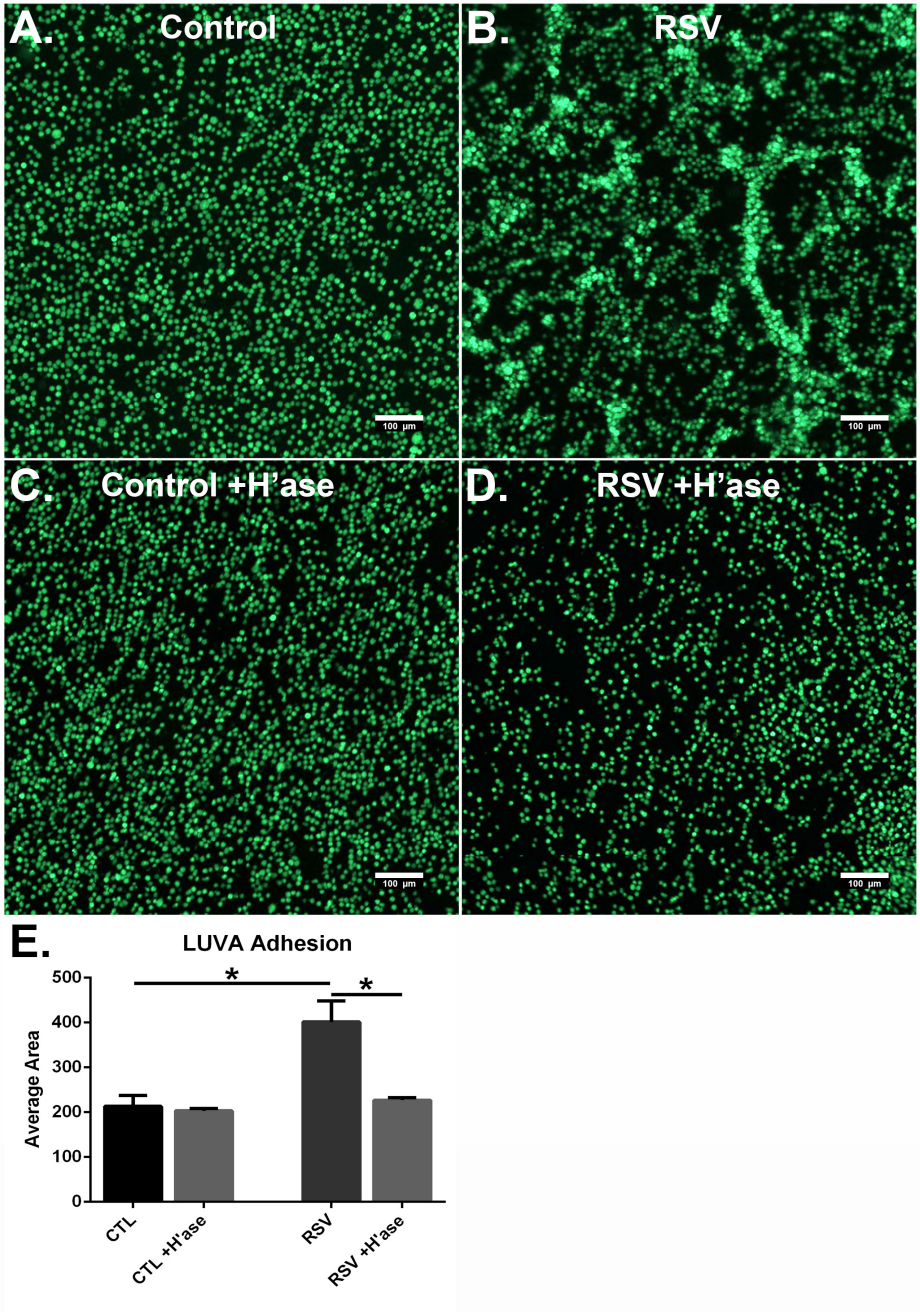
Adhesion of LUVA cells (green) to the ECM generated by **(A)** control HLFs compared to **(B)** RSV-infected HLFs. Note that LUVA cells aggregated in clusters in a pattern similar to that shown in Figure 2. Panels **(C,D)** depict LUVA binding following pre-treatment of the wells with *Streptomyces* hyaluronidase for control HLFs and RSV-infected HLFs, respectively. **(E)** Quantitative analysis of the staining area revealed a 2-fold increase in LUVA binding in adhesion studies conducted with RSV-infected HLFs compared to control HLFs (401.6 ± 18.94 units vs. 213.2 ± 9.818 units, ^*^P < 0.0001). The enhanced binding was eliminated in samples pre-treated with hyaluronidase (^*^P < 0.0001). Data shown as mean ± SD for *N* = 6 replicates/group.

To determine if the HA-enriched ECM produced by HLFs following RSV infection would promote greater adhesion of mast cells compared to that of control HLFs, adhesion of LUVA cells using quantitative fluorescent live-cell imaging was assessed. Compared to control HLFs (Figure 3A) adhesion of LUVA cells to the ECM generated by RSV-infected HLFs (Figure 3B) was significantly greater. The increased binding in RSV-infected conditions compared to controls was reversible by pretreating the wells with *Streptomyces* hyaluronidase (Figures 3C,D). Quantitative analysis of the staining area in each of these conditions revealed a 2-fold increase in LUVA binding in adhesion studies conducted with RSV-infected HLFs compared to control HLFs (401.6 ± 18.9 units vs. 213.2 ± 9.8 units, *P* < 0.0001). The enhanced binding was eliminated in samples pre-treated with hyaluronidase (*P* < 0.0001, Figure 3E).

**Figure 4:**
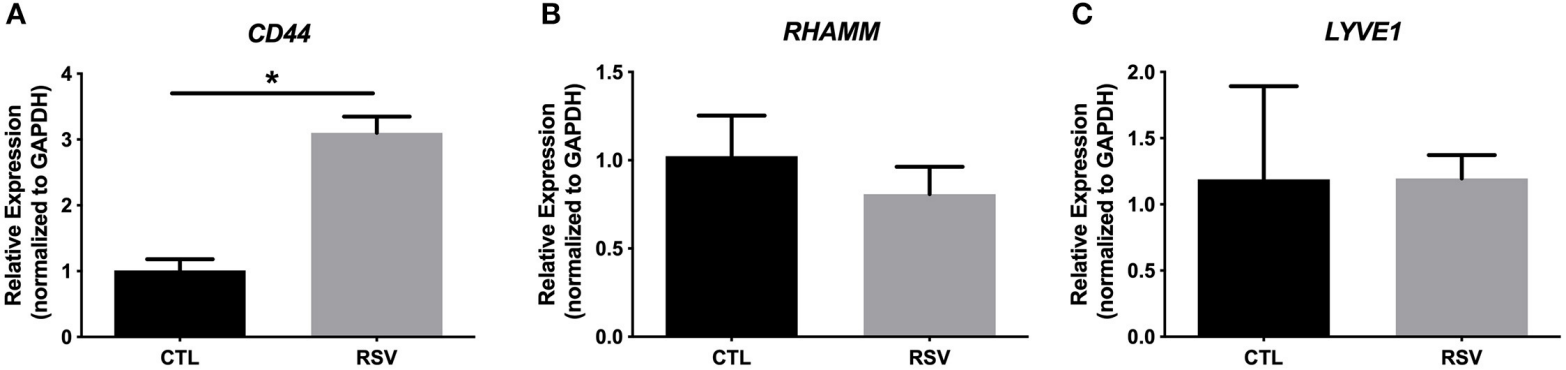
Gene expression of HA receptors by LUVA cells co-cultured with HLFs with or without RSV infection for 48 h. **(A)** LUVA cells co-cultured with RSV-infected HLFs demonstrated an increased expression of *CD44* mRNA compared to LUVA cells alone (^*^P = 0.002). No differences in LUVA cell expression of *RHAMM* or *LYVE1* were observed **(B,C)**. Gene expression was normalized to GAPDH and is shown as normalized mean ± SD relative to control LUVA cells for *N* = 6 replicates/group.

**Figure 5:**
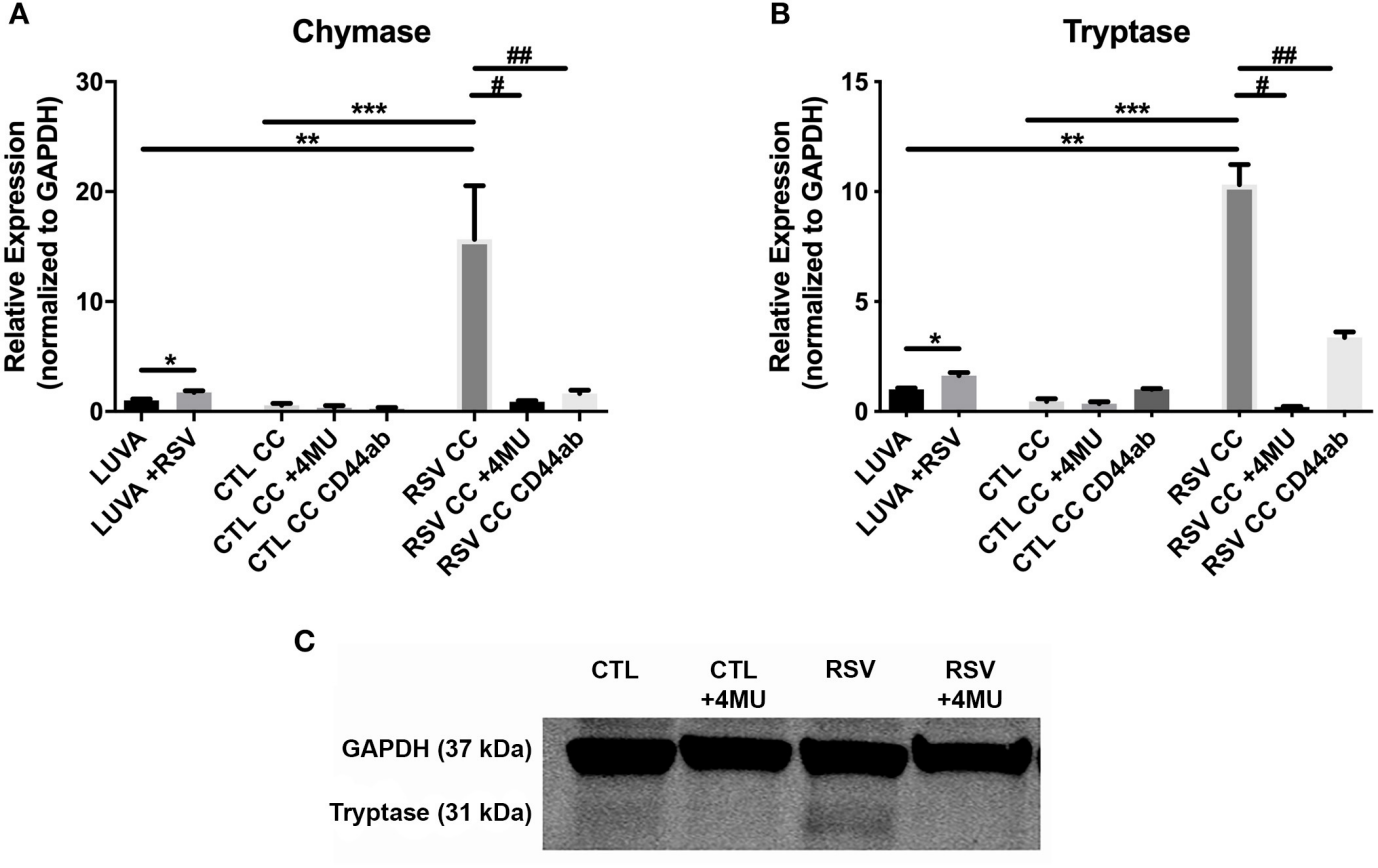
Gene expression of mast cell proteases by LUVA cells co-cultured (CC) with HLFs with or without RSV infection for 48 h (RSV CC and CTL CC, respectively). **(A)** LUVA cells co-cultured with RSV-infected HLFs demonstrated an increased expression of chymase mRNA compared to LUVA cells alone (^**^P = 0.01) and LUVA cells co-cultured with uninfected HLFs (^***^P = 0.002). RSV infection of LUVA cells alone did lead to a small increase in chymase expression at baseline (^*^1.7-fold, *P* = 0.03). Treatment with either 4-MU or neutralizing antibody to CD44 significantly decreased chymase expression by LUVA cells co-cultured with RSV-infected HLFs (^#^*P* = 0.02; ^*##*^*P* = 0.01, respectively). **(B)** Expression of tryptase by LUVA cells co-cultured with RSV-infected HLFs was increased compared to LUVA cells alone (^**^P = 0.01) and LUVA cells co-cultured with uninfected HLFs (^***^P = 0.002). RSV infection of LUVA cells alone led to a slight increase in tryptase expression at baseline (^*^1.7-fold, *P* = 0.03). Treatment with either 4-MU or neutralizing antibody to CD44 decreased the expression of tryptase by LUVA cells co-cultured with RSV-infected HLFs (^#^*P* = 0.02; ^*##*^*P* = 0.01, respectively). **(C)** Tryptase protein assayed by western blot confirmed a similar pattern as the gene expression analysis. Gene expression was normalized to GAPDH and is shown as normalized mean ± SD relative to control LUVA cells for *N* = 3–6 replicates/group.

To assess whether the interactions with the ECM generated by RSV-infected HLFs would lead to alterations in mast cell phenotype beyond increased adhesion, co-cultures of LUVA cells with HLFs following the establishment of the HA-enriched ECM for an additional 48 h were performed. LUVA cells were then isolated and assayed for expression of mast cell proteases as well as expression of HA receptors. Expression of the three major HA receptors (*CD44, RHAMM*, and *LYVE1*) was evaluated by PCR. Co-culture with RSV infected HLFs led to a 3-fold increase in CD44 expression by LUVA cells (*P* = 0.002, Figure 4A) without significant changes in RHAMM or LYVE1 expression (Figures 4B,C). To determine the role of HA in the induction of protease expression, a condition with the HAS inhibitor 4-MU as well as a condition using a monoclonal neutralizing antibody to the HA receptor, CD44, were included (Figure 5). LUVA cells co-cultured with RSV-infected HLFs exhibited an increased expression of chymase mRNA compared to LUVA cells alone (*P* = 0.01, Figure 5A) or to LUVA cells co-cultured with uninfected controls (*P* = 0.002). RSV infection of LUVA cells alone lead to a small increase in chymase expression at baseline (1.7-fold, *P* = 0.03); however, the magnitude of this increase is small in comparison to the HLF/RSV-infected co-culture condition. Pre-treatment with the HAS inhibitor 4-MU decreased the expression of chymase by LUVA cells co-cultured with RSV-infected HLFs to levels similar to LUVA cells alone (*P* = 0.02). Addition of monoclonal anti-CD44 antibodies also significantly attenuated the expression of chymase by LUVA cells co-cultured with HLFs following RSV infection (*P* = 0.01). LUVA cells co-cultured with RSV-infected HLFs also demonstrated an increased expression of tryptase mRNA compared to LUVA cells alone (*P* = 0.01) or to LUVA cells co-cultured with uninfected controls (*P* = 0.002, Figure 5B). Similar to chymase mRNA expression, RSV infection of LUVA cells alone led to a slight increase in tryptase expression at baseline (*P* = 0.03). Additionally, pre-treatment with 4-MU also decreased the expression of tryptase by LUVA cells co-cultured with RSV-infected HLFs (*P* = 0.02). Similarly, the addition of monoclonal CD44 blocking antibodies significantly attenuated the expression of tryptase by LUVA cells co-cultured with HLFs following RSV infection (*P* = 0.01). Tryptase expression assayed by western blot confirmed a pattern of protein expression that was similar to the gene expression analysis (Figure 5C).

### Impact of RSV on the Expression of Hyaladherins

**Figure 6:**
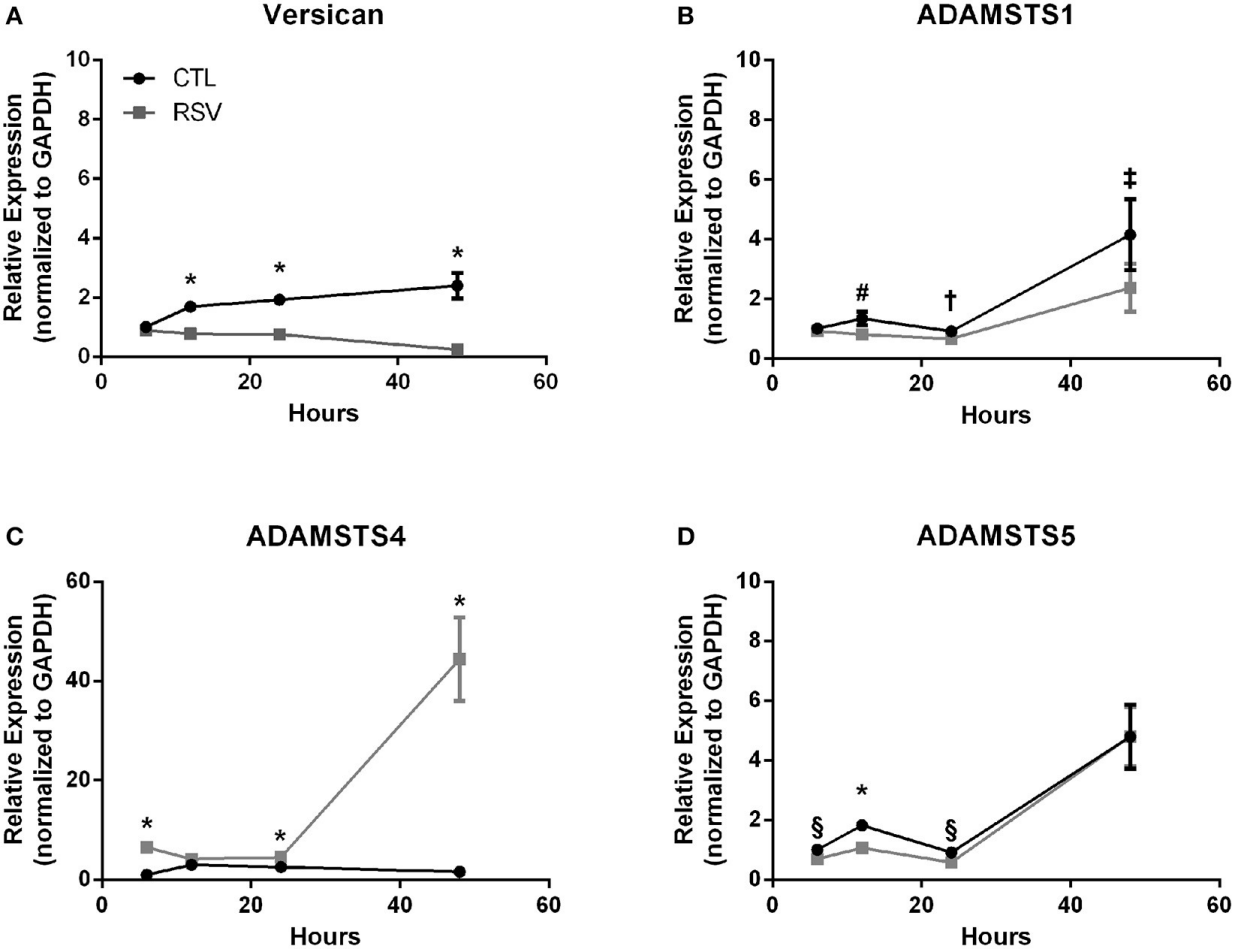
Time course of versican and versicanase expression by HLFs following infection with RSV. **(A)** Beginning at the 12-h timepoint, versican gene expression was decreased in RSV-infected HLFs compared to control HLFs (*P* > 0.0001, each comparison). **(B)** No significant differences were observed for ADAMSTS1 gene expression between the groups at 6 h. Expression of ADAMSTS1 was decreased at 12 h (^#^*P* < 0.005), 24 h (^†^*P* < 0.05), and 48 h (^‡^*P* < 0.002) following RSV infection. **(C)** Gene expression of ADAMSTS4 was significantly increased at 6 h, 24 h, and 48 h in RSV-infected HLFs compared to control HLFs (^*^P < 0.0001, each comparison). **(D)** Gene expression of ADAMSTS5 was significantly decreased in RSV-infected HLFs at 6 h (^§^*P* < 0.01), 12 h (^*^P < 0.0001), and 24 h (^§^*P* < 0.01), but not at the 48-h timepoint.

**Figure 7:**
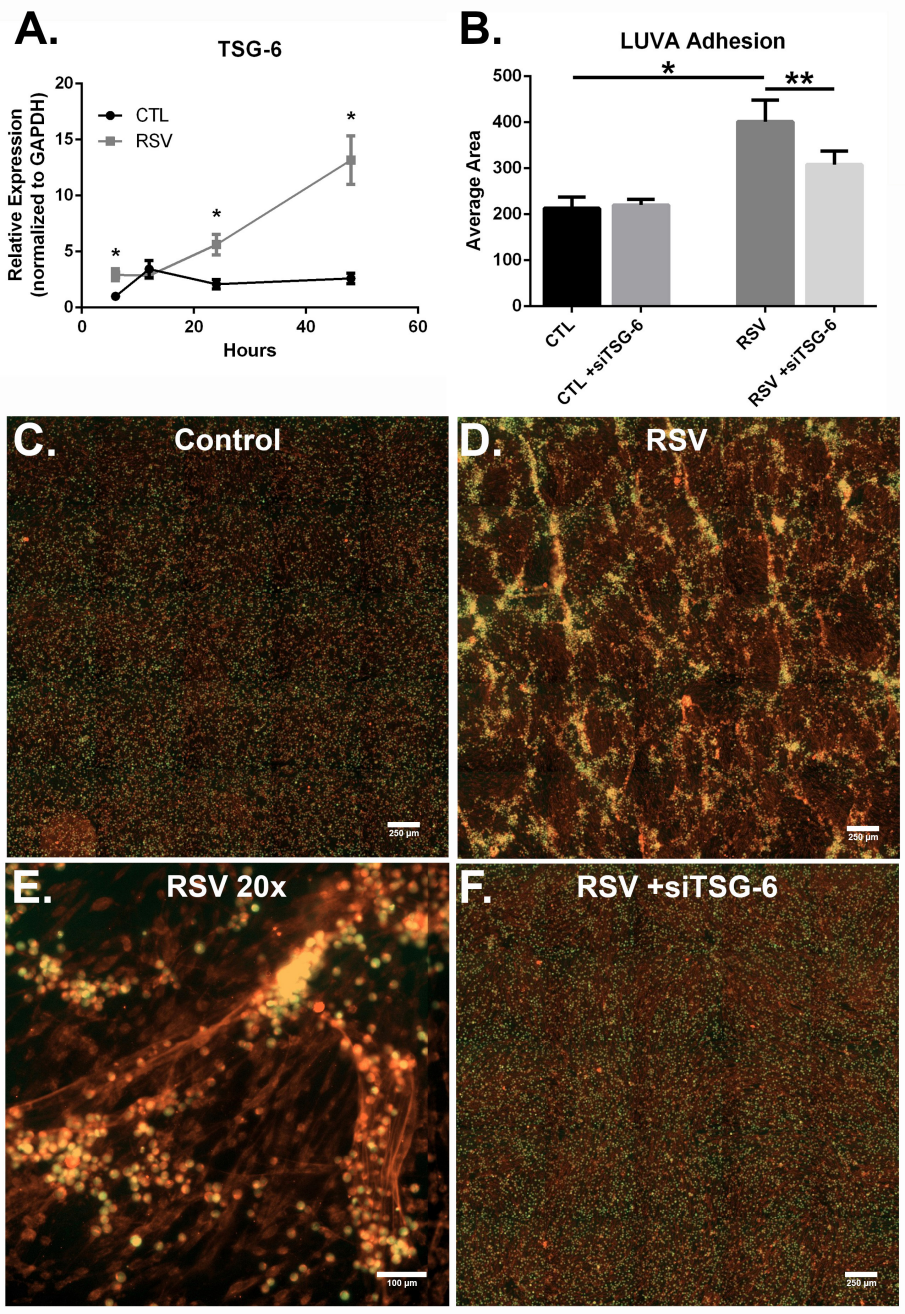
The effect of TSG-6 expression by HLFs following RSV infection on mast cell binding. **(A)** TSG-6 expression by HLFs was significantly increased following 6, 24, and 48 h of RSV infection (^*^P < 0.0001, each comparison). Gene expression was normalized to GAPDH and is shown as normalized mean ± SD relative to control HLFs at 6 h. **(B)** Quantitative analysis of LUVA cells following knockdown of TSG-6 in a live-cell binding assay revealed a significant reduction in the binding of LUVA cells observed in HLF following RSV infection (54% reduction, ^**^P < 0.002). Immunohistochemistry of adherent LUVA cells (green) to the HA ECM (red) generated by **(C)** control HLFs compared to **(D)** RSV-infected HLFs. Binding of LUVA cell aggregates is demonstrated in the RSV-infected HLF cultures along HA cable structures **(E)**. Aggregates of bound LUVA cells decreased following pre-treatment with siRNA to inhibit TSG-6 expression **(F)**. Data shown as mean ± SD for *N* = 6 replicates/group.

Previous work with HLFs demonstrated an important role for versican (VCAN), a large chondroitin sulfate proteoglycan that interacts with HA, in the enhanced binding of leukocytes following the application of the viral mimetic poly I:C (17). Thus, we examined the role of VCAN in our model system as well. We found that *VCAN* mRNA expression by HLFs was significantly suppressed following a 48-h infection with RSV (10-fold decrease, *P* < 0.0001, Figure 6A). HLF gene expression of versicanases (ADAMSTS1, ADAMSTS4, and ADAMSTS5) was elevated following the 48-h RSV infection (Figures 6B–D). Given the decreased VCAN synthesis and enhanced expression of VCAN degradation enzymes it seems unlikely that VCAN is driving the HA related effects in this model system. In contrast, we observed a 6-fold increase in the expression of mRNA for the hyaladherin TSG-6 in RSV-infected HLFs by 48 h (*P* < 0.0001, Figure 7A). Given that TSG-6 has been shown to be an important mediator of HA cable formation and HA-dependent inflammatory changes (30), and that it plays a critical role in the establishment of airway inflammation in murine models (31), we hypothesized that TSG-6 may also be contributing to the enhanced HA-dependent mast cell binding that we have demonstrated.

To determine the role of TSG-6 in our model, we repeated the mast cell adhesion assays following siRNA knockdown of TSG-6 expression in control and RSV-infected HLFs. This led to a significant reduction in the HA-dependent binding of LUVA cells observed in the RSV-infected HLF condition (54% reduction, *P* < 0.002, Figure 7B). Following live-cell adhesion assays, samples were fixed, stained, and re-examined for HA staining (representative images shown in Figures 7C,D). Wells from the RSV-infected HLFs demonstrated increased HA accumulation with LUVA cells embedded in the HA-enriched ECM (Figure 7D). Closer inspection revealed that LUVA cells were associated with dense cable-like structures of HA (Figure 7E) and that siRNA knockdown of TSG-6 expression prevented this association, contributing to the decreased binding of LUVA cells in that condition (Figure 7F).

**Figure 8:**
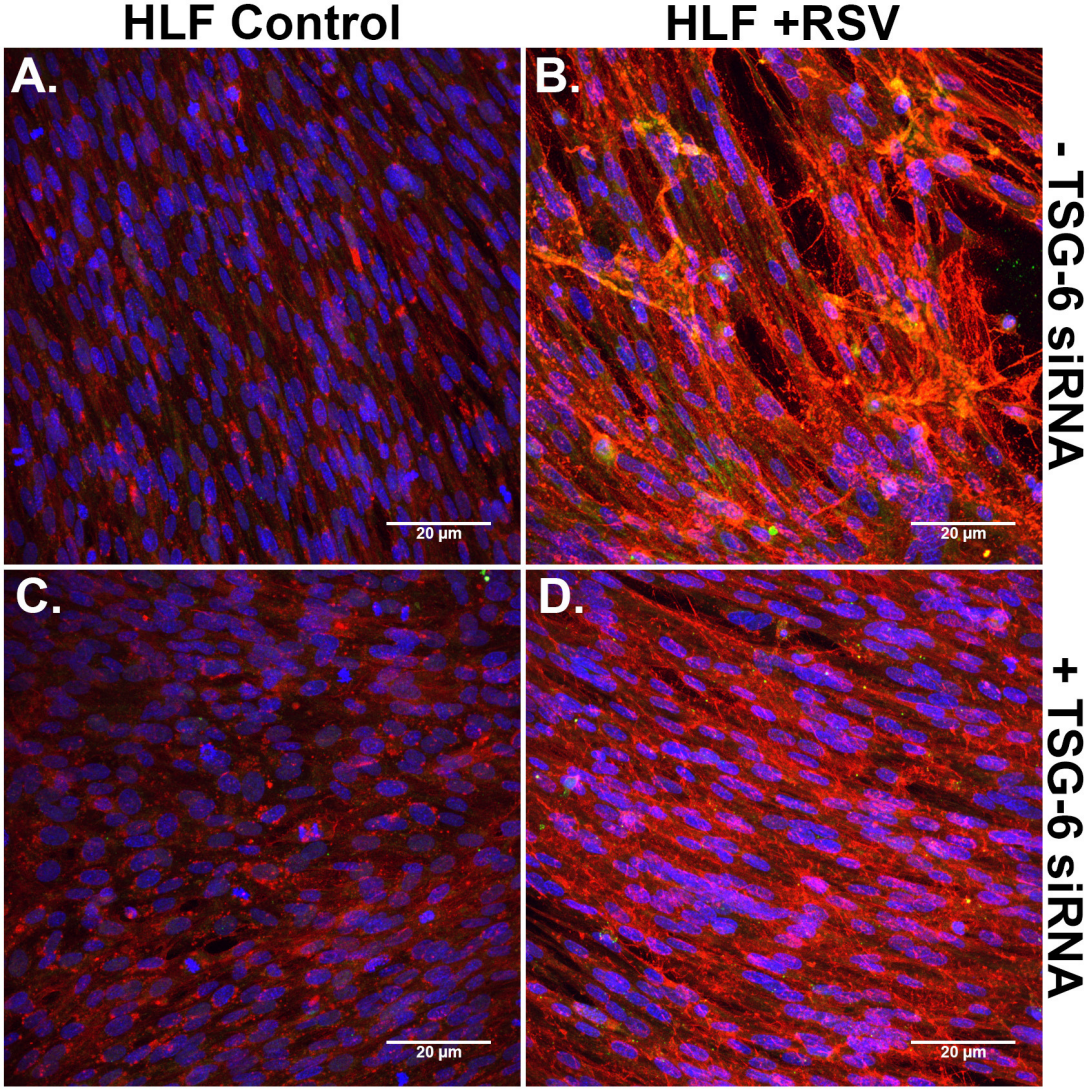
Immunohistochemistry for HA (red) and ITIH1, i.e., HC1 (green), accumulated in the ECM generated by HLFs following a 48-h RSV infection. **(A)** Uninfected control HLFs displayed a modest amount of HA production and relatively little incorporation of ITIH1 into the ECM. **(B)** RSV-infected HLFs demonstrated increased HA staining and organization of the ECM in higher order structures that also stained positively for ITIH1. Inhibition of TSG-6 expression did not alter the pattern observed in control conditions **(C)**; however, inhibition of TSG-6 expression decreased the amount of staining for ITIH1 and decreased the number of higher order HA structures observed in the RSV-infected HLFs **(D)**.

Immunofluorescent staining for HA and ITIH1, a heavy chain (HC) covalently bound to HA via TSG-6 activity, in the ECM by HLFs during control conditions demonstrated a modest amount of HA production and relatively little incorporation of ITIH1 into the ECM (Figure 8A). Conversely, RSV infected HLFs demonstrated increased HA staining and organization of the ECM in higher order structures that stained positively for ITIH1, i.e., HC-HA (Figure 8B). Inhibition of TSG-6 expression did not alter the pattern observed in control HLFs (Figure 8C); however, inhibition of TSG-6 expression decreased staining for ITIH1 and decreased the presence of higher order HA structures observed in the RSV infected HLFs (Figure 8D).

**Figure 9:**
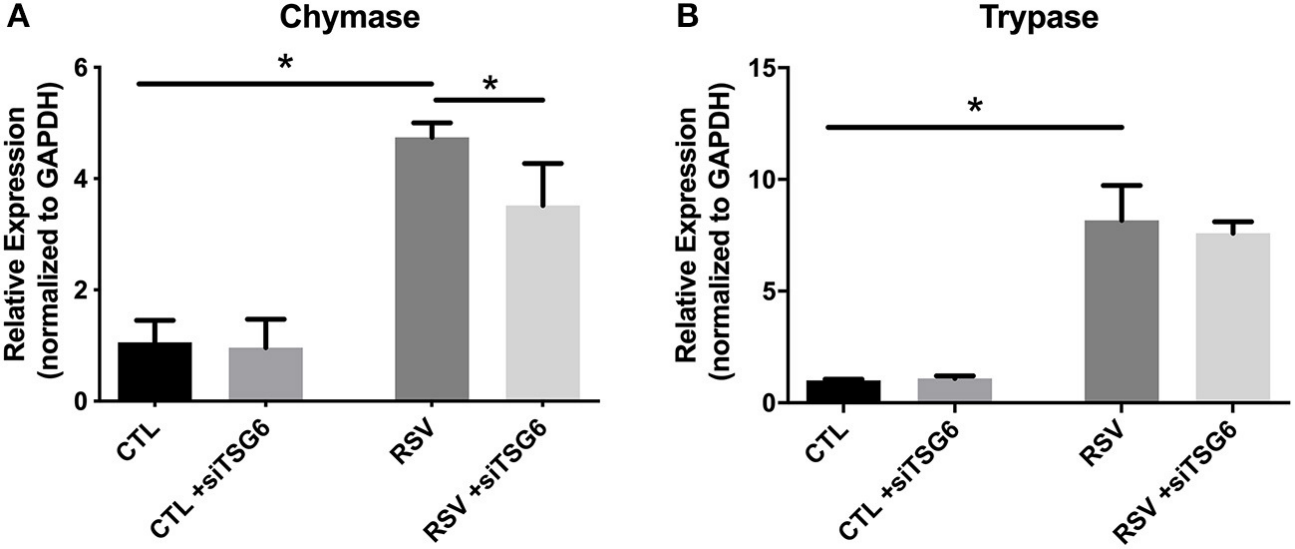
Gene expression by LUVA cells co-cultured with RSV-infected HLFs with or without knockdown of TSG-6 expression by siRNA. **(A)** LUVA chymase expression was increased following co-culture with RSV-infected HLFs (^*^P = 0.03) but was found to be significantly reduced by treatment of HLFs with TSG-6 siRNA (^*^P = 0.03), HLFs (^*^P = 0.03). **(B)** Expression of tryptase was also increased following co-culture with RSV-infected HLFs (^*^P = 0.03); however, it was not significantly different following treatment of HLFs with TSG-6 siRNA. Gene expression was normalized to GAPDH and is shown as normalized mean ± SD relative to control HLFs for *N* = 4 replicates/group.

Finally, we examined the role of TSG-6 knockdown on mast cell protease expression by the LUVA cells after a 48-h co-culture period. In these studies, we observed a modest 1.3-fold reduction in chymase expression (*P* = 0.03) and no significant changes in tryptase expression (Figure 9).

## Discussion

**Figure 10:**
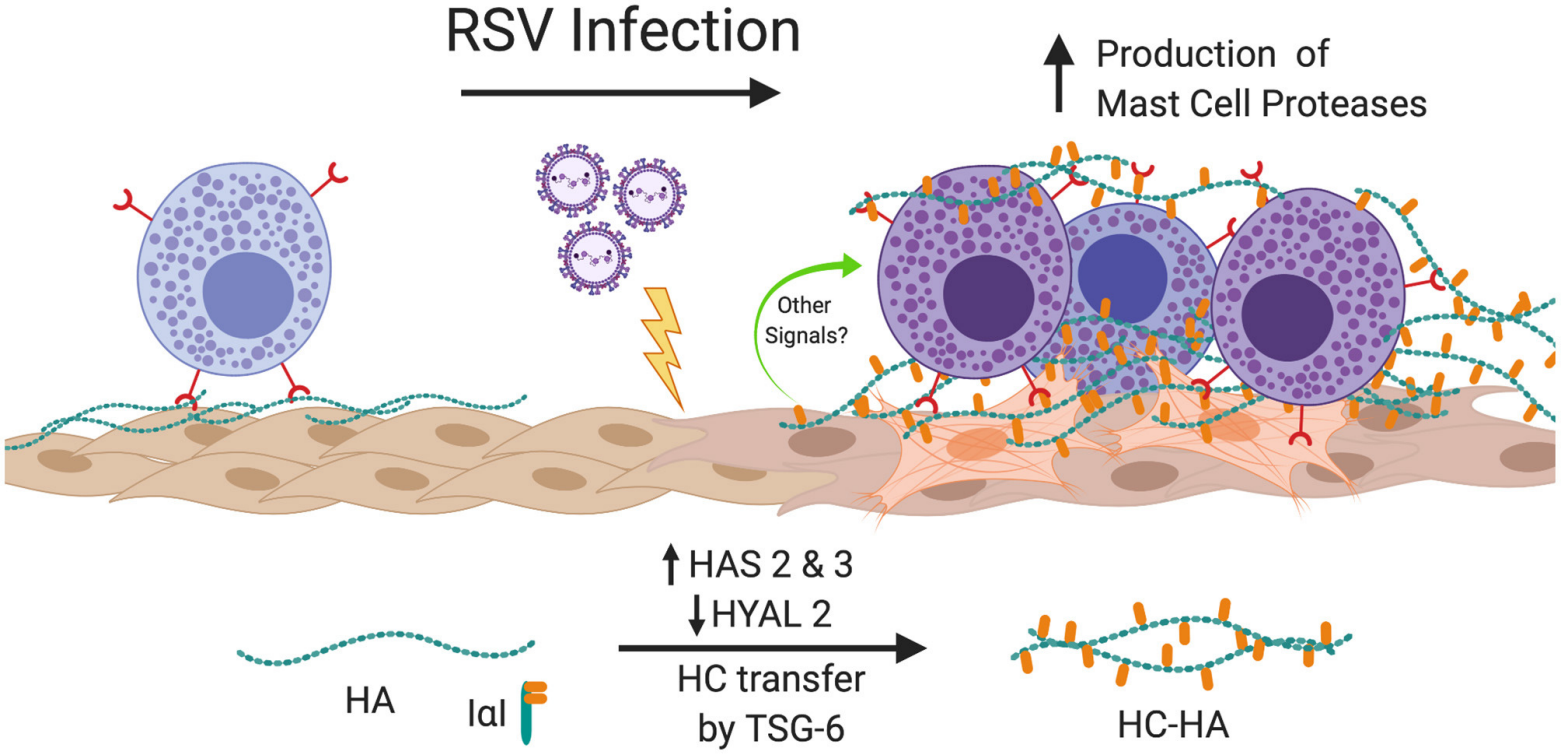
Summary of findings. In this study, we have shown that infection of HLFs with RSV induces upregulation of HAS2 and HAS3 with concomitant downregulation of HYAL2 leading to greater accumulation of HA in the HLF cell layer that displays greater adherence of mast cells via direct interactions with the HA-enriched ECM via a CD44 dependent mechanism. Furthermore, RSV infected HLFs display increased expression of TSG-6, which in turn catalyzes the transfer of ITIH1 to form HC-HA and enhances the binding of mast cells to the ECM. Mast cells bound to the RSV-induced ECM upregulate expression mast cell proteases contributing to the proinflammatory milieu. Blocking the formation of the HA-enriched ECM or blocking the CD44 receptor with neutralizing antibodies attenuates this process. Figure created with BioRender.com.

The findings of the present study demonstrate for the first time that RSV infection of primary pediatric donor-derived HLFs produces an ECM that is enriched with HA that is more adhesive for mast cells. The accumulation of HA in the cell layer was accompanied by the enhanced expression of HAS2 and HAS3 while HYAL2 expression was decreased in RSV infected HLFs. Furthermore, we have demonstrated that this binding is accompanied by augmented expression of the mast cell proteases, chymase and tryptase. The increased mast cell binding was dependent on the presence of HA following specific digestion of the ECM by hyaluronidase. Furthermore, inhibition of HA-enriched ECM formation through the use of 4-MU, an inhibitor of HA synthesis, or blockade of the HA receptor CD44 with monoclonal antibodies, demonstrated that enhanced mast cell protease expression was dependent on the interaction with this HA-enriched ECM. Additional studies to evaluate the contributions of the hyaladherin, TSG-6, demonstrated that knockdown of TSG-6 expression partially decreased enhanced binding of the LUVA cells to the ECM and partially decreased the expression of chymase, but did not alter expression of tryptase. These data implicate the formation of an HA-enriched ECM in promoting a proinflammatory milieu during acute respiratory viral infections (Summary Figure 10).

Mast cells have been long recognized to play a critical role in allergic airway inflammation and asthma (32, 33); however, their contributions to innate immunity and host defense during acute respiratory infections have also been increasingly recognized (34, 35). Unlike circulating white blood cells, mast cells mature in tissues and the local microenvironment plays a critical role in the differentiation of mast cells and the establishment of their phenotype. Serine proteases are unique to mast cells and have been recognized for their contributions in several inflammatory diseases including asthma (36). Furthermore, the expression of proteases by mature mast cells is shaped by local environmental factors (35). Previous studies have demonstrated an important role for interactions with HA via the CD44 receptor during proliferation and differentiation of mast cells (23, 37, 38), thus for these studies we chose to investigate the effect of interacting with the RSV-induced ECM in LUVA cells, a human mast cell line (24). Other studies have examined the direct effects of RSV infection of mast cells and shown that inflammatory cytokines such as type-I interferon production is significantly increased; however, mast cell protease production was not assessed (39). In our experiments, LUVA cells alone did display slight increases in chymase and tryptase following RSV exposure, but the magnitude of this increase was relatively small compared to that displayed by LUVA cells cultured on the HA-enriched ECM generated by RSV-infected HLFs. While we cannot rule out that signaling mediators produced by HLFs may contribute to the enhanced protease production by the LUVA cells, the reduction in protease expression following the inhibition of the HA-enriched matrix produced by HLFs with 4-MU would suggest a direct effect of this ECM in driving this process. Furthermore, inhibition of modifications to HA by TSG-6, i.e., HC-HA, via siRNA knockdown suggests an important role for HA in either directly signaling to the LUVA cells or holding them in close apposition to the HLFs so that other direct or indirect signaling may occur. These findings are interesting in that we are demonstrating for the first time that ECM induced by RSV infection of HLFs can alter the expression of inflammatory mediators by mast cells. It is possible that other proinflammatory mediators expressed by mast cells, as well as IgE-dependent and IgE-independent stimuli of mast cells are altered by the interaction with this “pathological” HA matrix; however, examination of these factors is beyond the scope of this report and will be an interesting area for future investigation.

The role of HLFs in establishing a HA-enriched, proinflammatory ECM has been previously explored (17). In those studies, sub-confluent primary HLFs treated with poly I:C produced an ECM that was enriched with HA and VCAN that led to increased binding of a human monocyte cell line (U937 cells). Furthermore, treatment with poly I:C led to a shift in the HA content from the culture media to the cell layer and an increase in the hydrodynamic size of the HA (i.e., HMW-HA), but only a modest increase in the total amount of HA accumulation in the HLF cultures. Interestingly, Potter-Perigo et al. found that the increased accumulation of HA and VCAN was secondary to relative decreases in degradation pathways (17). While there are several similarities between the findings of that study and the findings that we report herein, there are also several important differences. In the present study, HLFs were infected with RSV capable of replication resulting in a sustained infection of the HLFs which may lead to a very different exposure profile compared to single-dose poly I:C exposure. We also sought to examine VCAN expression in our model system and found that it was significantly reduced in HLFs following a 48-h RSV infection. Thus, VCAN expression was unlikely to explain the enhanced mast cell binding that was observed in the present study.

In a more recent study, Gaucherand et al. demonstrated that co-culture of HLFs with activated T lymphocytes produced an HA-enriched, leukocyte adhesive ECM (11). Similar to our findings, the authors reported a decreased expression of versican along with an increase in versican degrading enzyme expression. Additionally, that study reported increased expression of TSG-6 by HLFs following co-culture with activated T lymphocytes leading to the hypothesis that increased TSG-6 expression was contributing to the enhanced retention of monocytes in leukocyte binding assays. Similar to our data, HLFs co-cultured with the activated T lymphocytes produced a greater amount of total HA, which was largely retained in the cell layer (11).

A similarity between our studies is that TSG-6 seems to be an important player in the establishment of the leukocyte-adhesive ECM. Previous work has demonstrated an important role for TSG-6 in promoting the crosslinking of HA into larger cable-like structures (30, 40). Separate studies have also identified an important role for TSG-6 in other models of lung inflammation, including asthma and allergen challenge (41, 42). Additionally, the presence of TSG-6 has been shown to enhance the production of HA-enriched matrices and increase the ability of these matrices to adhere leukocytes (43). Knockdown of TSG-6 expression in our system led to a significant reduction in the retention of LUVA cells; however, this did not decrease LUVA cell binding to the degree that digestion of the matrix HA content with hyaluronidase did, suggesting that while TSG-6 is important for LUVA cell binding in our system, other factors may also contribute.

In another recent study from our group, we have reported that co-culture of primary airway epithelial cells (AECs) obtained from asthmatic pediatric donors with healthy donor-derived primary HLFs led to increased synthesis of an ECM that was enriched with HA and led to increased retention of leukocytes compared to co-culture of HLFs with AECs derived from healthy donors (12). That study taken together with the findings of the present study and the studies discussed above (11, 17) reveal some important similarities about the behavior of HLFs despite exposure to somewhat different pro-inflammatory conditions. In each of these studies, expansion of the amount of HA contained within the cell layer as well as a shift toward increased HMW-HA species were present. Although what causes the retention of the HMW-HA in the cell layer in these experiments is unknown, one possible explanation is that the HA is somehow being protected from degradation and/or turnover by the presence of hyaladherins such as TSG-6 or HA modifications such as HC-HA. While this hypothesis remains to be directly tested in HLFs, it is possible that this could be an important mechanism for the establishment of pro-inflammatory or “pathological” HA matrices that promote lung inflammation given the elegant series of studies that have implicated TSG-6 as an important mediator of inflammation and airway hyperresponsiveness in asthma (15, 31, 42–44).

While the present study has several strengths, there are also some important limitations to consider. We have demonstrated that increased HA accumulation following RSV infection of HLFs is accompanied by increased transcription of *HAS2* and *HAS3* mRNA with concurrent downregulation of *HYAL2* transcription. While the altered balance of HA syntheses and degradation may be sufficient to explain the greater accumulation of HA during RSV infection it is important to note that HASes are also subject to post-translational modifications that can affect their enzymatic activity, stability, and localization (45–47). Investigation of these mechanisms is beyond the scope of the present study but would be important considerations in future studies as these mechanisms may also be important targets for regulating the formation of a proinflammatory RSV-induced ECM. Similarly, signaling pathways proximal to the upregulation of HASes such as NF-κB signaling or transcription co-factors such as HAS2-AS1 have been explored in other models and may also be important mechanisms for the regulation of HAS expression and accumulation of HA in the microenvironment in our model (48). Future studies aimed at addressing these considerations are needed.

In conclusion, we have demonstrated that infection of healthy pediatric HLFs with RSV leads to the establishment of a HA-enriched ECM that not only displays enhanced binding of mast cells, but also directly alters the phenotype of the cells that are interacting with this matrix. Additional molecules such as TSG-6 may be important in the establishment of this matrix through the formation of HC-HA, thus implicating its activity during inflammatory processes during acute viral infections. These studies raise the question as to whether HA or TSG-6 may be targets for therapeutic intervention during acute viral respiratory illnesses; however, further work will need to be done to characterize their contributions in *in vivo* model systems.

## Data Availability Statement

The raw data supporting the conclusions of this article will be made available by the authors, without undue reservation, to any qualified researcher.

## Author Contributions

SR, AP, TW, and JD drafted/edited the manuscript. SR, IK, SZ, AP, TW, and JD designed experiments. KB, LR, MW, NS, and CC made significant contributions to experiments, maintenance of the model system, and data analysis.

### Conflict of Interest

The authors declare that the research was conducted in the absence of any commercial or financial relationships that could be construed as a potential conflict of interest.
